# Circular Shear Printing of Spiral-Oriented CF-PP Components for Enhanced Mechanical Performance and Warp Mitigation

**DOI:** 10.3390/polym17131739

**Published:** 2025-06-22

**Authors:** Dashan Mi, Tao Yang, Jinghua Jiang, Haiqing Bai, Shikui Jia

**Affiliations:** 1 School of Mechanical Engineering, Shaanxi University of Technology, Hanzhong 723001, China; charlyang2@163.com (T.Y.); jjhsnut@163.com (J.J.); 2 School of Materials Science and Engineering, Shaanxi University of Technology, Hanzhong 723001, China; shikuijia@snut.edu.cn

**Keywords:** mechanical properties, high-shear printer, carbon fiber, orientation

## Abstract

Extrusion-based printers have attracted much attention for their simplified printing process and broader material compatibility. Carbon fibers (CF), known for their excellent mechanical properties, are incorporated into polypropylene (PP) printing materials. This study presents a shear screw printer (SSP) with a modified screw design. The SSP generates torsional shear forces, enabling helical orientation of CFs within PP/CF composites. The study also compares the SSP’s performance with that of a conventional screw printer (CSP). PP/CF composite specimens containing 15% CF were printed at four different layup angles: 0°, 45°, 90°, and ±45° (net). The results show that combining CFs’ helical orientation with a net printing arrangement can effectively enhance tensile properties while reducing anisotropy. Furthermore, this approach can significantly mitigate warping in printed parts.

## 1. Introduction

Additive manufacturing of polymers encompasses various methods, such as Selective Laser Sintering (SLS), Fused Deposition Modeling (FDM), and Stereolithography (SLA). Among these, FDM has become the most widely used due to its relatively low cost, benefiting from simple constructive solutions that are inexpensive to implement and easy to operate [1,2]. However, FDM requires the preliminary processing of pellets into filaments. These filaments are then extruded layer by layer onto the build platform, where they fuse to form the final object. While filament-based 3D printers nowadays can utilize a variety of polymers, the desire for novel materials and applications, faster deposition speeds, and lower costs have driven the development of alternative printing methods, such as Extrusion Additive Manufacturing (EAM) [3]. Similarly to industrial polymer extruders, EAM has evolved into various configurations including conical screw-based extrusion [4], single-screw printer [5], counter-rotating twin-screw printer [6], and co-rotating twin-screw printer [7], among others. EAM enables the use of innovative materials (e.g., micro- or nano-reinforced polymers), facilitates faster and more precise control over the extrusion process [8], and allows for partial material mixing [9]. Screw-assisted deposition tools are now advanced for applications in recycling, biofabrication, and personalized medicine [10,11].

Among the most commonly used thermoplastics, polypropylene (PP) stands out as a semi-crystalline polymer that is widely applied in consumer and technical products. This is attributed to its remarkable mechanical properties, simple processing requirements, and affordable price. However, when employed in 3D printing, PP exhibits significant shrinkage during cooling (attributed to its semi-crystalline structure), leading to warping and detachment from the printer bed [12]. This results in poor dimensional accuracy, failing to meet production requirements. To mitigate these issues, fillers such as amorphous polypropylene (aPP) have been introduced to reduce crystallinity and minimize geometric deformation [13]. Furthermore, the addition of rigid fibers like glass fiber (GF) or carbon fiber (CF) has been shown to substantially decrease warping [14]. Both long, short, and recycled CF variants have been extensively incorporated into 3D-printed parts. CF offers enhanced reinforcement due to its superior mechanical properties and high thermal stability, improving composite performance while reducing associated life-cycle costs and environmental impacts [15].

Although 3D-printed PP/CF composites have the aforementioned advantages, their mechanical properties are influenced by build orientation and raster pattern. This is largely attributable to the inherent characteristics of the layer-based process and the directional nature of raster patterns in 3D printing, which results in the samples displaying marked anisotropic behavior [16]. In the conventional extrusion printing process, the melt undergoes stretching after extrusion, and the nozzle geometry, especially the convergent angle and output diameter, greatly influences the flow behavior [17]. The fibers’ orientation in the deposited path aligns with the printing direction, which indicates improved mechanical properties along the printing direction [18].

In contrast, inspired by natural fibrous systems, Jordan R. [19] has created a rotational 3D printing technique that allows for the spatial control of short fiber orientation. Experimental outcomes indicate that this method, which combines parallel paths with rotated CF, can greatly boost tensile strength and modulus.

In this study, by optimizing the screw structure, we simplified the rotational 3D printing method and reduced the cost of printing equipment that provides torsional shear forces. To the best of our knowledge, there have been few studies on how different printing angles for spiral-oriented CF affect anisotropy and warpage. In this research, a homemade single screw with a special shear element is used to provide shear for deviating CF orientation, forming a circumferential CF orientation, which is expected to enhance the tensile performance in the circumferential direction and suppress part deformation. A comparative analysis is conducted with a conventional screw. Furthermore, different printing angles are applied to explore the role of CF helical orientation in reducing anisotropy and part warpage.

## 2. Experiment

### 2.1. Materials

iPP (trade name: PPH-T03) was obtained from Sinopec Corporation (Maoming, China). The composite masterbatch containing 70% iPP and 30% CF was purchased from Dongguan Yushuo New Materials Technology Co., Ltd. (Dongguan, China).

### 2.2. Sample Preparation

**Figure 1:**
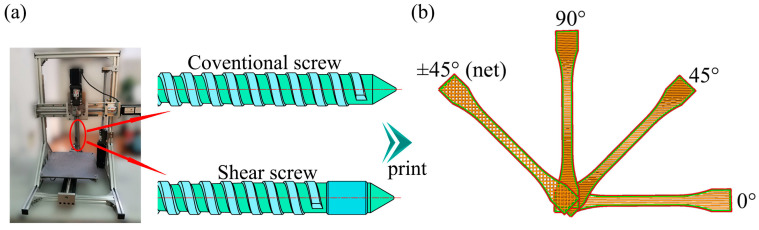
(**a**) Schematic of conventional screw and shear screw, (**b**) schematics of a dog-bone specimen with layup angles of 0°, 45°, 90°, and ±45° (net).

In this study, the screw extrusion-based 3D printer employs two types of screws, as shown in Figure 1a. The first type is the conventional screw (CS), with an outer diameter of 16 mm. The lengths of the feed, compression, and metering zones are set to 4, 6, and 5 times the outer diameter, respectively. The compression ratio ε (defined as the ratio of the depths of the feed and metering zones) is calculated using the following equation: [20]

(1) $$\varepsilon =\frac{h_{F}(t-s)(D-h_{F})}{h_{M}(t-s)(D-h_{M})}$$

where h_F_ is the depth of the screw groove in the feeding zone, while h_M_ is the depth of the screw groove in the metering zone. For both the conventional screw (CS) and shear screw (SS), D = 16 mm, h_F_ = 3.1 mm, and h_M_ = 0.8 mm, resulting in a compression ratio ε = 3.29. A printer that uses this type of screw is called a conventional screw printer (CSP).

The shear screw (SS) incorporates shear elements in the melting zone of the screw, with a clearance of 75 μm between the shear elements and the barrel wall. This design provides high shear to achieve helical orientation of carbon fibers (CF). The printer using this screw is called the shear screw printer (SSP).

PP pellets or PP/CF blends are fed directly through the hopper and printed in four different layup angles, as shown in Figure 1b. All samples are printed with a bed temperature of 120 °C and an extruder temperature of 200 °C. The nozzle diameter is set to 0.5 mm, and the final printing layer thickness is adjusted to 0.5 mm. The screw rotation speeds for CSP and SSP are 10 rpm and 20 rpm, respectively. Since high CF content may block the 3D printing nozzle, while low CF content would weaken the reinforcement effect, this study selects 15% CF blends as the research subject [21]. Sample naming conventions are detailed in Table 1. For example, “CF-SSP90” indicates a sample with 15% CF content, printed using SSP with a printing orientation of 90°.

### 2.3. Mechanical Properties

The dumbbell-shaped specimens used for the tensile test (30 mm × 5 mm × 2 mm for the narrow section) were printed according to ISO 527-2:2012E [22]. Tensile tests were conducted at an ambient temperature (20 °C) using an electro-mechanical universal testing machine (GOTECH-20KN, Guangdong GOTECH Testing Instruments Co., Ltd., Dongguan, China) with a crosshead speed of 5 mm/min. Property values were taken as the average of five specimens.

### 2.4. Crystal Structure

The melting characteristics of the samples were analyzed via Differential Scanning Calorimetry (TA DISCOVERY DSC2500, TA Instruments, New Castle, DE, USA) in a nitrogen environment. The samples underwent heating at 10 °C/min from 40 to 200 °C, were maintained at 200 °C for 3 min, and subsequently cooled to 40 °C at 10 °C/min. The degree of crystallinity (X_c_) was assessed using XRD (Bruker D8 Advance, Bruker Corporation, Billerica, MA, USA) and calculated with the following equation [23]:

$$X_{c}=\frac{A_{c}}{A_{c}+A_{a}}$$

Here, A_c_ is the fitting intensity of the crystallization peaks, and A_a_ is the corresponding intensity of the amorphous phase.

### 2.5. Morphology

Scanning electron microscopy (SEM, ZEISS Gemini 300 SEM, Carl Zeiss AG, Germany) was used to analyze the fracture surface morphology after tensile testing. Before the analysis, the fracture surfaces were gold-sputtered. The SEM images were obtained using voltages of 3.0 kV and 5.0 kV. Additionally, the orientation of carbon fibers (CFs) was examined using optical microscopy (OM, MSD1125, Murzider Technology Co., Guangzhou, China) following the heating and melting of samples on a hot stage. X-ray-computed tomography (XCT, NanoVoxel1000, Sanying Precision Instruments Co., Ltd., Shanghai, China) was performed on the composites, with a standard measurement resolution of 3 μm/voxel.

## 3. Results and Discussion

### 3.1. Crystal Structure

**Figure 2:**
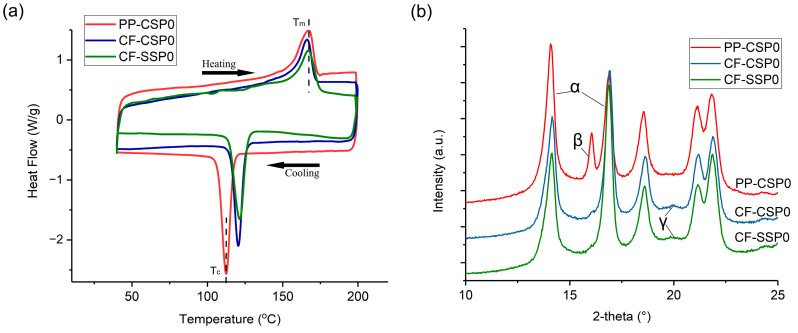
(**a**) DSC heating and cooling curves, (**b**) XRD curves.

Figure 2 compares the DSC and XRD profiles of PP-CSP0, CF-CSP0, and CF-SSP0; the key thermal and structural parameters are summarized in Table 2. These three formulations isolate the effects of carbon-fiber (CF) addition and of processing in conventional-screw (CSP) versus single-screw (SSP) printers. After adding CF to pure PP, the melting-onset temperature (T_m onste_) slightly increased from 154.2 °C for PP-CSP0 to 156.7 °C for CF-CSP0. This suggests that CF addition optimizes the crystal structure’s integrity and improves the thermal stability. In the cooling segment of Figure 2a, CFs serve as nucleating sites, causing the crystallization peaks to shift significantly to the right. As a result, compared to PP-CSP0, the crystallization-onset temperature (T_c onset_) of CF-CSP0 increases by 7.8 °C. Furthermore, since shear stress can enhance nucleation density and rate [24], CF-SSP0, with higher shear force, further improves the ability of CF to nucleate, increasing the T_c onset_ by an additional 1.6 °C. Meanwhile, CF addition raised the crystallization temperature by about 10 °C, allowing PP more time to crystallize during printing, and thus increasing the final degree of crystallinity (X_c_). The X_c_ of PP-CSP0 was only 39.6%, but with CF addition, X_c_ rose to 65.2% for CF-CSP0, and further to 67.1% for CF-SSP0. That is to say, CF addition can promote heterogeneous nucleation. Moreover, SSP, with its shearing effect, can orient CFs, further enhancing their heterogeneous nucleation and increasing the degree of crystallinity. XRD (Figure 2b) reveals that CF addition suppresses the β-phase and promotes γ-phase formation in PP. The augmented X_c_ and altered crystal polymorphism are expected to bolster tensile strength (see Section 3.2).

### 3.2. Effects of High Shear and CF Addition on Mechanical Properties

**Figure 3:**
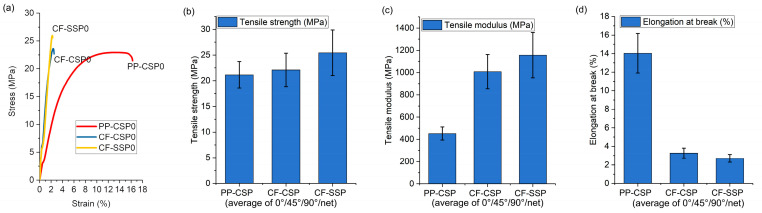
Tensile properties averaged over 0°, 45°, and 90° and net layup angles: (**a**) selective stress–strain curves, (**b**) tensile strength, (**c**) tensile modulus, (**d**) elongation at break.

This study examined the tensile behavior of specimens printed at various orientations to assess the combined effects of carbon fibers (CF) and screw-induced shear. The results in Figure 3 are averages values of all printing angles. In Figure 3a, representative stress–strain curves reveal that CF incorporation markedly increases both strength and stiffness, albeit at the expense of ductility. Figure 3b shows that combining CF with SSP increases tensile strength from 21 MPa (PP-CSP) to 25 MPa (CF-SSP), with reasonable error ranges of 1.9 to 2.6. Figure 3c highlights a more significant increase in tensile modulus. The modulus of PP-CSP is 452 MPa, but CF addition boosts it by 137%, to 1008 MPa for CF-CSP, mainly because CF hinders deformation. Using SSP further increases the modulus to 1157 MPa. This is partly because SSP’s high shear improves CF dispersion [9], reducing stress concentration points, and partly because shear aligns CF. In addition, shear can promote CF-induced crystallization. When fibers are extracted from the melt or when shear stress is applied at a specific crystallization temperature, transcrystalline structures form. The growth of transcrystalline layers on carbon fibers strengthens polymer–fiber interactions, thereby boosting mechanical strength [25,26]. Finally, Figure 3d shows that CF’s rigidity reduces overall fracture strain: both CF-CSP and CF-SSP exhibit elongations at break of approximately 3%.

### 3.3. Effects of CF Orientation on Mechanical Properties

The orientation of carbon fibers (CF) is primarily determined by the screw rotation shear and the straight shear from the extrusion nozzle wall during the printing process. Additionally, different orientations of CF can affect the mechanical properties of the printed parts.

**Figure 4:**
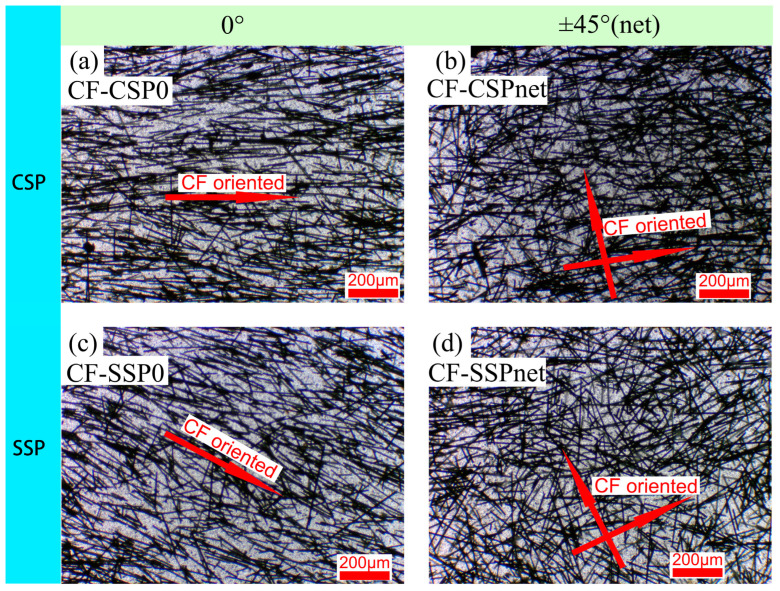
Light microscopy of PP/CF nanocomposites (**a**) CF-CSP0, (**b**) CF-CSPnet, (**c**) CF-SSP0, (**d**) CF-SSPnet.

As shown in Figure 4a, when printed with a conventional screw printer (CSP), carbon fibers (CFs) tend to align along the printing direction due to the straight shear from the extrusion nozzle wall. The shear rate at the nozzle wall can be calculated as follows [27]:

$$\dot{\gamma }=\frac{4\cdot Q}{\pi \cdot R^{3}}$$

Q represents the volumetric flow rate = 1.96 mm^3^/s, and R represents the nozzle radius = 0.25 mm (since diameter is 0.5 mm). Substituting the values into the formula gives a shear rate of 160 s^−1^. This result is similar to other researchers’ reports of similar shear rate magnitudes, at approximately 300 s^−1^ [28]. The shear prompts CFs to align along the printing path. As in Yan et al.’s study [29], the extrusion width impacts fiber orientation, with a linear correlation between them. With the 0.5 mm extrusion width used here, fibers are expected to exhibit a more uniform alignment. With net printing, CSP can form grid-like structures with a ±45° (net) arrangement, as shown in Figure 4b.

**Figure 5:**
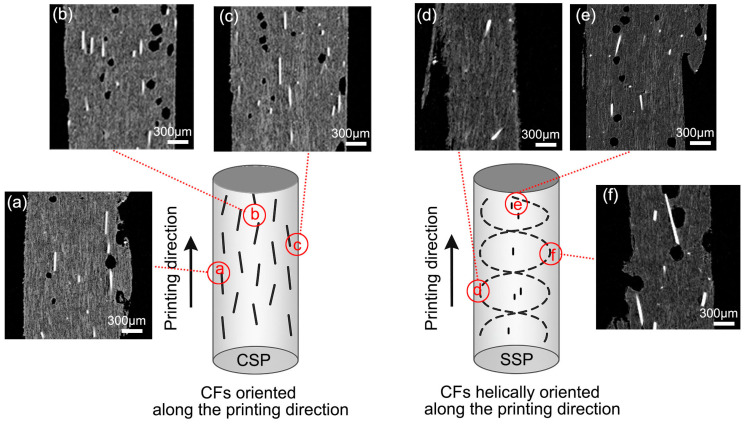
Schematic of CF orientation and XCT images of CF-CSP0 (**a**–**c**) and CF-SSP0 (**d**–**f**).

In SSP, CFs experience more complex shear forces from the shear element and nozzle. Inside the screw, CFs are subjected to circumferential shear stress caused by the shear elements. As this shear device is located near the extrusion end, it is expected to retain the shear effect on the extruded melt. The shear rate can be calculated using the following formula [30]:

$$\dot{\gamma }=\frac{\pi D_{A}N}{\delta }$$

where D_A_ is the external diameter of the actual screw (16 mm), N is the rotation speed of the screw (20 rpm, 0.33 rps), and δ is the clearance between the screw and the barrel (δ = 0.075 mm). Substituting the values into the formula results in a shear rate of 221 s^−1^.This shear causes the CFs to adopt a circular orientation. Combined with the straight shear from the nozzle wall, most fibers ultimately align in the direction of the arrow in Figure 4c, which is at an angle to the printing direction. Figure 5 will probe into the specific deflection angle using XCT.

Figure 5 further verifies the morphology of CFs in CSP and SSP via XCT. For CSP (Figure 5a–c), the stretching shear during extrusion causes CFs to align with the printing direction at different positions, exhibiting a parallel orientation consistent with the CSP schematic.

As for SSP (Figure 5d–f), CFs show a hybrid morphology of partial parallelism and deflection. This is due to the gradient of the circumferential shear stress from the shear element, which causes inconsistent deflection in CFs while making most of them deflect. This leads to a deflection angle between the CFs and the printing direction of 10° to 45°. However, the cross-sectional plane examined by XCT cannot accurately represent the orientation angles of carbon fibers (CFs), which are primarily aligned in the circumferential direction. Therefore, it provides a qualitative indication of CF deflection. The following text will elaborate on how this CF deflection impacts mechanical properties.

**Figure 6:**
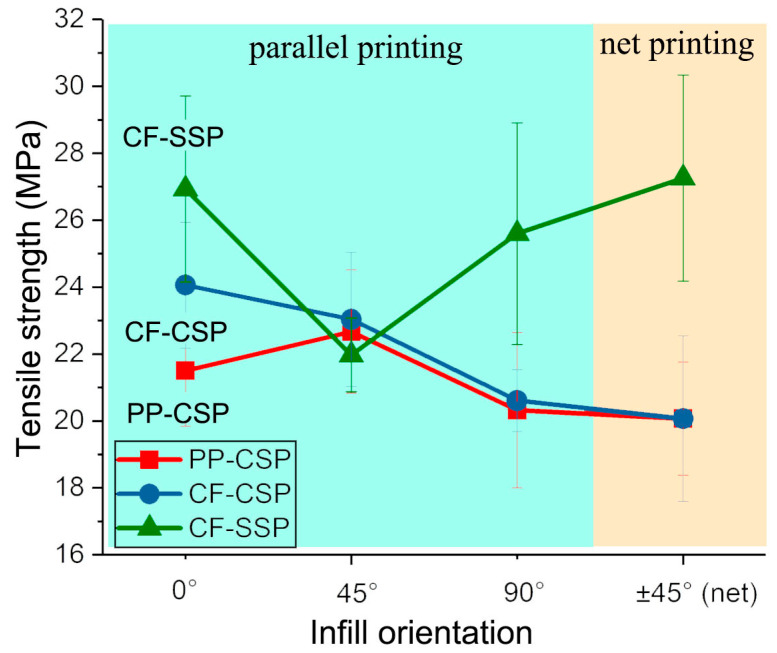
Tensile strength of PP-CSP, CF-CSP, and CF-SSP by different printing orientations.

Figure 6 compares the tensile strength of PP-CSP, CF-CSP, and CF-SSP at different printing angles (0°, 45°, and 90°), which correspond to specimens being stretched in different directions. PP-CSP, without CF addition, exhibits poor mechanical properties. The tensile strength of CF-CSP at 0° is 12% higher than that of pure PP-CSP, reaching 27 MPa. However, as the printing angle deviates from 0° to 90°, the reinforcing effect of CF is compromised. For CF-CSP90, the fibers and matrix align perpendicular to the printing direction. This particular orientation results in significant interlayer separation and a marked reduction in mechanical performance. The tensile strength of CF-CSP90 drops to 23 MPa, close to pure PP (PP-CSP90). The standard deviation of CF-CSP90 is 0.92, slightly lower than PP-CSP90’s 2.32.

For SSP, the tensile strength of CF-SSP0 is 25% and 12% higher than that of PP-CSP0 and CF-CSP0, respectively. This shows that even when carbon fibers (CFs) are no longer parallel to the force direction after high shear, the shear-induced uniform CF orientation, along with higher crystallinity and better CF dispersion, still boosts the tensile strength. Additionally, the spiral shear is expected to enhance the interlayer interface bonding strength by altering the molecular chain entanglement state [31]. As observed by Ning et al., high-speed rotary shearing can increase the tensile strength of parts printed at 0° by about 50%. This is largely due to the higher speed of the external shearing element. In our study, the shearing elements integrated on the screw must align with the screw speed for printing, which results in a relatively lower speed.

However, our approach has still achieved commendable results in maintaining the strength of prints at both 0° and 90° orientations. For instance, the tensile strength of CF-SSP90 reaches 26 MPa, which is on par with that of CF-SSP0. However, for the 45° printing direction, the tensile strength of CF-SSP45 significantly drops to 22 MPa, which is 18% lower than CF-SSP0. This indicates a change in fiber orientation within the melt, altering the part’s strength distribution. It seems that with SSP, CFs may have achieved a 45° orientation. When combined with a 45° printing angle, the CFs become perpendicular to the stretch direction, reducing the tensile strength.

This also indicates that when printing fiber-reinforced melts in a parallel-aligned manner, there is still a specific angle at which the mechanical properties are significantly inferior, regardless of whether the fiber orientation changes from shear extrusion. This variation in mechanical properties at different stress angles is termed anisotropy. The following formula can be used to quantify the anisotropy coefficient (R_aniso_):

$$R_{aniso}=\frac{T_{max}-T_{min}}{T_{max}}\times 100\%$$

where T_max_ and T_min_ are the maximum and minimum tensile strengths at different printing angles, respectively. The value of R_aniso_ ranges between [0, 1), with 0 indicating isotropy and values closer to 1 reflecting greater degrees of anisotropy.

In this study, when using a parallel layup arrangement, the R_aniso_ values for PP-CSP, CF-CSP, and CF-SSP are 10%, 14%, and 18%, respectively. This indicates that PP-CSP exhibits the lowest degree of anisotropy, but also exhibits the lowest mechanical properties. Although CF-SSP effectively enhances tensile strength through carbon fiber (CF) reinforcement, it results in significant anisotropy. This anisotropy can limit the practical application of printed parts. Therefore, using a parallel printing approach only allows for a choice between high strength and low anisotropy. To achieve a balance, a net printing angle is required.

A net printing angle can reduce the anisotropy from parallel layups but fails to enhance the tensile strength of CF-CSP. In net-style printing, the molten material forms point-to-point contact at the interface (Figure 1b). This contact has a smaller area than the line contact in parallel-printed parts. Additionally, the increased void content at the interlacing points inhibits effective stress transfer, thus reducing the tensile strength. Consequently, the tensile strength of PP-CSPnet and CF-CSPnet is only 20.1 MPa, lower than that of other parallel-printing samples. However, CF-SSPnet exhibits 36% higher tensile strength than PP-CSPnet and CF-CSPnet, with a standard deviation of 3, which is within a reasonable range. Its strength matches CF-SSP0 and ensures uniform multi-directional mechanical strength. It maintains high tensile strength while eliminating anisotropy in the printing plane. The following text will explore why CF-CSPnet achieves such a good performance from fiber orientation and fracture modes. The other tensile properties, like tensile modulus and elongation at break, are briefly given in Figure S1 as Supplementary Information.

### 3.4. Effects of CF Orientation on Fracture Surface

**Figure 7:**
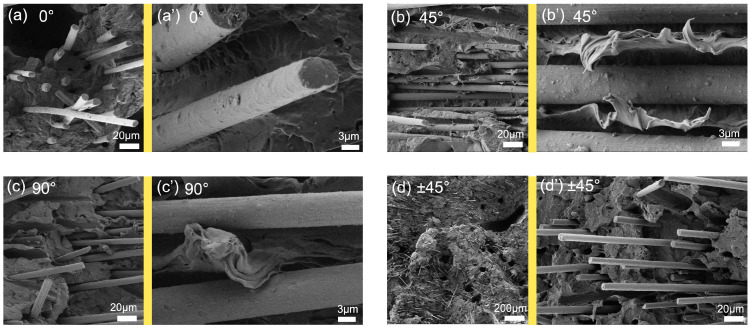
SEM images of tensile fracture surface: (**a**) CF-SSP0, (**b**) CF-SSP45, (**c**) CF-SSP90, and (**d**) CF-SSPnet.

To verify that fiber orientation affects mechanical properties, Figure 6 shows the tensile fracture morphologies of SSP samples at different printing angles. At 0° (Figure 7a), many fibers protrude from the fracture surface matrix. This indicates sliding between the matrix and fibers or CF breakage during loading. The latter absorbs external energy, and more extensive fiber breakage results in higher average tensile strengths and modulus [32].

At 45° (Figure 7b′), most fibers are parallel to the fracture surface, and the matrix undergoes plastic deformation perpendicular to the CFs, indicating that many fibers have deflected at 45°. Here, the CFs neither break nor slide relative to the matrix, which reduces energy absorption. Consequently, CF-SSP45 exhibits the lowest tensile strength and stiffness among the SSP samples.

At 90° (Figure 7c), some CFs in CF-SSP90 protrude from the matrix, and fibrillation and fibril-bridging toughening are observed at the crack tip [33]. Other CFs appear parallel to the fracture surface (Figure 7c′), as the oriented fibers prevent horizontal matrix fracture, inclining the fracture surface and enhancing mechanical properties, as will be later detailed.

At ±45°, CF-SSPnet shows a very rough fracture surface. To better illustrate this, Figure 7d uses a smaller magnification. Although some CF orientations in CF-SSPnet match those in CF-SSP45, the complex CF orientation structure inclines the fracture surface. This causes fibers of various orientations to protrude from the matrix (Figure 7d′), allowing them to absorb energy during tension. Consequently, CF-SSPnet achieves the highest tensile strength and modulus.

**Figure 8:**
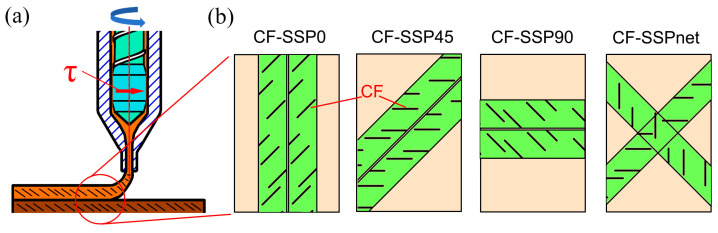
Schematic of (**a**) CF orientation during printing, and (**b**) CF orientation at different printing angles.

Based on the fracture morphologies discussed above, Figure 8 presents a schematic diagram of fiber orientation within SSP samples. As shown in Figure 8a, the rotating screw’s shear elements generate a circumferential shear rate τ (221 s^−1^). When CFs pass through a 75 µm slit, they orient circumferentially. Subsequent stretching during extrusion combines with this orientation to produce the final CF orientation. As shown in Figure 8b, various CF orientations are displayed. Given that XCT can only qualitatively indicate CF deflection—yet combined with the fact that in the tensile fracture surface of the CF-SSP45 sample, numerous CFs are parallel, and considering that the part manufactured at 45° printing has the worst tensile properties—it can be inferred that SSP causes CFs to deflect at nearly 45°. Thus, the CF orientation schematic in Figure 8b is obtained.

At 0° printing, CFs form a 45° angle with the part’s lengthwise direction.

At 45° printing, CFs are perpendicular to the lengthwise direction, making them less effective in reinforcing tension. This explains the minimal tensile strength and modulus of CF-SSP45.

At 90° printing, CFs again form a 45° angle with the lengthwise direction.

At net printing, CF orientations are partially perpendicular or parallel to the lengthwise direction.

In summary, CFs in SSP are not parallel to the printing direction due to shear-induced orientation. The following text will compare the fracture surface of CSP and SSP, highlighting the deviated CF effects on fracture surface.

**Figure 9:**
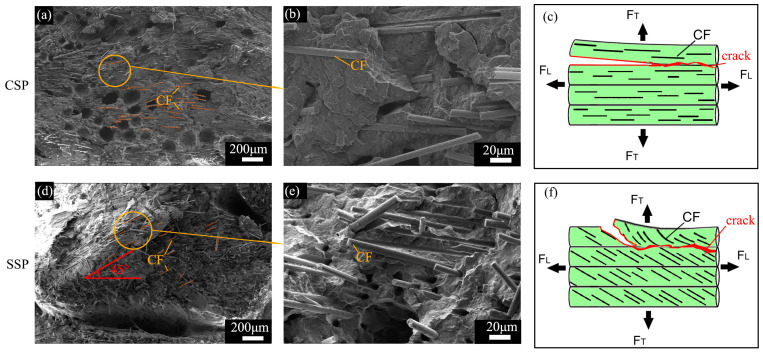
SEM image and schematic of tensile fracture surface of (**a**–**c**) CF-CSP90 and (**d**–**f**) CF-SSP90.

In this study, samples with a printing angle of 90° from CSP and SSP were chosen to observe the effect of CF deviated orientation on tensile fracture morphology, as the printing direction is perpendicular to the loading direction. Figure 9 shows the fracture surfaces of CF-CSP90 and CF-SSP90, with CFs marked by dashed lines for better visibility. Overall, CSP exhibited a relatively smooth fracture surface with most fibers arranged horizontally and parallel to the matrix. As illustrated in Figure 9c, when CFs are aligned with the printing direction, the longitudinal force (FL) can be transferred to the CFs. However, as the applied force gradually shifts to the transverse direction (FT), the fibers become perpendicular to the force direction. Cracks propagate along the weld line, resulting in a smooth and horizontal tensile fracture surface, indicating that CFs cannot effectively enhance the material in this orientation.

In contrast, the fracture surface of SSP was rough, with the left side significantly higher than the right side. CFs were observed to be inserted into the matrix at an approximate 45° angle. As shown in the magnified view (Figure 9e), the fracture surface was covered with fibers that protruded from the matrix. These fibers prevented the matrix from fracturing horizontally, instead forming a 45° ridge. Such a fracture structure significantly enhances tensile strength and toughness. As depicted in Figure 9f, when the force direction is perpendicular to the layup direction, the fracture surface propagates along the CFs due to their deviated oriented arrangement. This allows the CFs to participate in the tensile energy absorption process, thereby improving the tensile strength.

### 3.5. Reduction in Warpage by CF Helical Orientation

**Figure 10:**
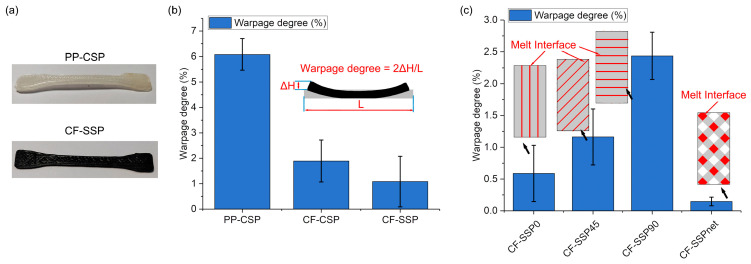
(**a**) Image of selected warped samples, (**b**) averaged warpage degree of PP-CSP, CF-CSP, and CF-SSP, (**c**) warpage degree of CF-SSP in various layup angles and schematic of melt interface.

Figure 10a shows the warpage of printed parts after cooling. PP-CSP exhibits significant warpage, while CF-SSP shows no visible warpage. Warpage was quantified using the formula below:

$$Warpage\;degree=2\times \frac{\Delta H(height\;of\;warp\;increase)}{L(original\;length)}\times 100\%$$

This formula is designed for strip-shaped samples. When a sample is placed on a flat surface and its ends curl upward or downward, the formula can be applied. However, if a sample exhibits irregular warpage like twisting, the formula cannot accurately represent the warpage extent. In this study, the samples display only one type of warpage, so the formula is suitable for quantification.

ΔH is the height increase due to warpage, and L is the original length of the part. The warpage degree ranges from [0, 1], with 0 indicating no warpage and 1 indicating maximum warpage.

Figure 10b shows that PP-CSP has the highest warpage of 6.1% when ignoring printing-angle effects. Adding carbon fibers (CFs), which provide a rigid skeleton, reduces warpage to 1.9% for CF-CSP. Other researchers have found that rigid fibers, such as glass fiber, can reduce the deformation of printed parts from 58 mm to 8 mm [14]. They also observed that increasing the content of rigid fibers, like glass fibers, can further suppress deformation [34]. In this study, without altering the fiber content but only modifying the fiber orientation, we have also optimized the degree of warpage. Specifically, the CF-SSP configuration has reduced warpage to 1.1%. This indicates that the helically distributed CFs in CF-SSP enhance both longitudinal and transverse modulus, suppressing warpage. The large error bars for CF-SSP in Figure 10b stem from its varying warpage characteristics across different printing angles.

Figure 10c illustrates SSP warpage at various printing angles. Warpage mainly occurs along the melt interface. For CF-SSP0, where the melt interface is longitudinally aligned, warpage is minimal at 0.6%. However, as the melt interface orientation shifts to transverse, warpage increases to 2.44% for CF-SSP90 due to weaker interlayer bonding. In contrast, CF-SSPnet transforms interface contact from linear to point-based. This, combined with the helical CF orientation, significantly reduces warpage, lowering it to 0.1%.

## 4. Conclusions

This study successfully fabricated PP/CF composites through SSP and CSP at four specific printing angles: 0°, 45°, 90°, and ±45° (net). In CSP, CFs aligned with the printing direction, whereas in SSP, the high shear force from shear elements induced a spiral CF orientation. Parallel printing angles (0°, 45°, and 90°) showed clear anisotropy, with R_aniso_ values of 10% (PP-CSP), 14% (CF-CSP), and 18% (CF-SSP). However, by combining spiral-oriented CFs with ±45° (net) printing, CF-SSPnet significantly mitigated in-plane anisotropy. Furthermore, CF-SSPnet achieved the highest tensile strength (27.3 MPa) and modulus (1323 MPa), as well as the lowest warpage (0.1%). This approach of integrating spiral-oriented carbon fibers (CFs) with net-style printing efficiently produces PP/CF components. These components not only exhibit high mechanical strength and improved shape stability, but also enable the printing of larger parts and enhance printing success rates. CF-SSPnet components, with their superior mechanical and shape-stable attributes, facilitate lightweight design and find broad applications across various fields, including aerospace (e.g., drone wings), mechanical manufacturing (e.g., robot arms), and sports equipment (e.g., bicycle frames).

Future research needs to further optimize the shearing element and minimize the shear-induced shortening of CFs. The goal is to achieve better mechanical properties and less warpage while preserving CF length as much as possible.

## Figures and Tables

**Table 1:** Sample naming conventions.

Sample	Printer	PP	CF	Printing Angle
PP-CSP0/45/90/net	Conventional screw printer	100%	0	0/45/90/net
CF-CSP0/45/90/net	Conventional screw printer	85%	15%	0/45/90/net
CF-SSP0/45/90/net	Shear screw printer	85%	15%	0/45/90/net

**Table 2:** Characteristic properties obtained by DSC: melting temperature T_m_, onset melting temperature T_m onset_, crystallization temperature T_c_, onset crystallization temperature T_c onset_, and crystallinity X_c_ determined by XRD.

Sample	T_m onset_ (℃)	T_m_ (℃)	T_c onset_ (℃)	T_c_ (℃)	X_c_
PP-CSP0	154.2	167.2	117.1	112.4	39.6%
CF-CSP0	156.7	166.2	124.9	124.9	65.2%
CF-SSP0	156.4	167.1	126.5	121.4	67.1%

## Data Availability

The original contributions presented in this study are included in the article/supplementary material. Further inquiries can be directed to the corresponding authors.

## Supplementary Materials

The following supporting information can be downloaded at: https://www.mdpi.com/article/10.3390/polym17131739/s1, Figure S1: Tensile modulus and Elongation at break.
