# Supplementary material for: Circular Shear Printing of Spiral-Oriented CF-PP Components for Enhanced Mechanical Performance and Warp Mitigation
Source: Polymers (Basel). 2025 Jun 22;17(13):1739. doi: 10.3390/polym17131739 (PMC12252335; doi:10.3390/polym17131739)
Supplement: Supplementary file 1 [file polymers-17-01739-s001.zip › polymers-3699244-supplementary.pdf]

As shown in Figure S1a, the tensile modulus of the samples was enhanced by the addition of carbon fibers (CF). The modulus of CF-shear screw printer (CF-SSP) composites remained high at all printing angles except 45°, and CF-SSPnet achieved a maximum modulus of 1323 MPa. This indicates that the combination of helically oriented CFs and net printing significantly improves the tensile modulus. Meanwhile, Figure S1b presents the elongation at break for each sample, with CF-SSPnet showing the lowest value. This can be attributed to its high rigidity and tensile modulus, which lead to more brittle fracture characteristics under tension.

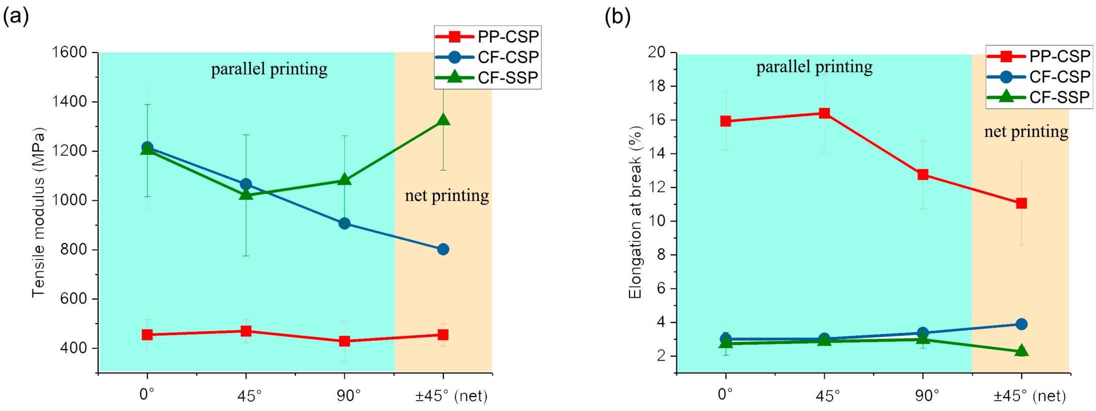

Figure S1. Tensile properties (a) Tensile modulus, (b) Elongation at break
